# Observations on Fumigation as a Therapeutic Agent

**Published:** 1824-11

**Authors:** J. Green


### Art. IX.—*Observations on Fumigation as a Therapeutic Agent.* By J. Green, Esq.

I am induced to offer you a short communication on the employment of the various fumigations, resulting from my experience since I adopted their administration as a branch of practice in Bury-street. I do not yet feel authorized to acquiesce with what has been so strenuously advanced by the continental writers, in favour of this remedy. Perhaps this may be owing to the inattention or reluctance with which some medical men in this country have regarded the subject, and from the imperfect trials which others are induced to give it.

This mode of bathing generally requires perseverance, and, like other baths, too, the benefit derived is not evinced until some time has elapsed after their discontinuance. In some instances, however, the contrary is the fact, and its agency has been so immediately beneficial, as to occasion surprise both to patient and physician; and, in other cases, not evidently dissimilar, its effects have not been shown so speedily.

Fumigations, though originally intended for the cure of diseases of the skin only, have, by subsequent experience, been found equally efficacious in a multiplicity of complaints, varying much in their nature, principally chronic; but of late acute diseases have been submitted with success to this remedy. In such cases, however, this being an exciting agent, previous depletion and medical treatment is indispensable. In chronic cases, it is frequently relied on alone: nevertheless, appropriate medicines, conjointly used, never fail to act with more certainty, even in smaller doses, and materially expedite the cure. The patients hitherto received here have been almost exclusively those of my medical friends, who have conducted the medical treatment: others have been placed more immediately under my own care by those friends, many of whom are ready to testify, that, when this remedy is properly administered, persevered in, and assisted by the usual adjuncts, that to speak of it in terms of moderation only, would be unjust.

Passing over skin-complaints, where the advantages cannot be denied, their usefulness in no cases have been more satisfactorily evinced than in congestions of the viscera, as in torpor of the liver: here the chlorine fumigations have been of most decided advantage; their superiority over the nitro-muriatic acid bath will not admit of dispute. This is borne out, too, by a very able work, lately published, see "Researches on Chlorine," by Wm. Wallace, of Dublin, who, in some obstinate skin-diseases, is inclined to give it the preference even to sulphureous fumigations. As far as my opinion goes, I have thought the occasionally substituting one for the other has been attended with the same good effects, as surgeons frequently experience by alternating the use of escharotics to an ulcer.

In cases of visceral obstruction or congestion, the relief obtained may be accounted for, from the increased flow of blood which the temperature of the bath is brought to circulate in the extreme vessels on the surface of the body; and, as this impetus is simultaneously given to the parts internally, local obstruction may be thus overcome. As there is no dense medium in this bath, as is the case in a water-bath, the weight of which, whilst in the bath, is sufficient to prevent the distention of the capillary vessels; an equalization of the circulation is effected, the blood being forced to flow in channels which have, perhaps, been for years with some persons almost impervious, particularly with those who have dry, arid, unperspiring skins.

On the same rationale, too, may perhaps rest a great deal of the benefit that is derived in skin complaints, independent of the medicinal substances used in the fumigation: for the cutaneous surface being by this means forced to assume a new and increased action, is enabled to throw off much by transpiration, that might otherwise be a remote or proximate cause of disease.

Following up a late pathology of rheumatism arising not from an increased action of the parts affected, but from a weakened and distended state of the vessels, this remedial agent must appear obviously advantageous: and that such may be the cause of the disease, no specious arguments are required to prove: witness the frequent turgescence of the vessels of the conjunctiva, the weakened and distended varicose veins in many parts of the body, with many persons; and, when these are situated in places subject to much motion, surrounded by hard substances, bones, tendons, and ligaments, as in the joints, such a view of the cause of rheumatism at least seems reasonable, and is borne out more strongly when it is considered that heat, redness, or inflammation, are not usual marks of rheumatism.

In affections of the lymphatic system, as in scrofulous swellings and ulcers, the principles of fumigation will appear obviously beneficial; so in all other affections depending on a weakened action in the vascular system. Nor is it one of the least boasts of this remedy, that it can be used in cases of extreme debility, as such patients commonly find their health improve whilst using the remedy, but more after a series of them have been taken, and then discontinued. Nor must its sovereign deserts be omitted here, in those troublesome cases the sequelæ of syphilis, or in those arising from the abuse of mercury. Its good effects are not less conspicuous in contractions and enlargements of the joints.

The writer's view in this communication is not so much to particularize the various complaints for which this remedy is indicated, as to point out its usefulness in a multiplicity of chronic and anomalous diseases, which are usually baffling to medical men, and where such simple, though important, means hold out a fair promise of a more successful mode of treating such complaints: at least, it cannot be denied that it is an additional remedy, at once powerful and simple, that should be added to the usual remedial powers we already possess. To introduce here cases corroborating what has been now advanced, is unnecessary: suffice it to say, they have been the patients of men of talent, and of justly acknowledged professional liberality, and who are disposed to appreciate duly the advantages of this plan of treatment.

